# Serial album validation for promotion of infant body weight control

**DOI:** 10.1590/1518-8345.2194.2998

**Published:** 2018-05-17

**Authors:** Nathalia Costa Gonzaga Saraiva, Carla Campos Muniz Medeiros, Thelma Leite de Araujo

**Affiliations:** 1 PhD, RN, Hospital Universitário Lauro Wanderley (HULW), João Pessoa, PB, Brazil.; 2 PhD, Professor, Nursing Department, Universidade Estadual da Paraíba, Campina Grande, PB, Brazil.; 3 PhD, Full Professor, Nursing Department, Universidade Federal do Ceará , Fortaleza, CE, Brazil.

**Keywords:** Body Weight, Obesity, Child, Health Promotion, Educational Technology, Validation Studies

## Abstract

**Objective:**

to validate the content and appearance of a serial album for children aged from 7 to 10 years addressing the topic of prevention and control of body weight.

**Method:**

methodological study with descriptive nature. The validation process was attended by 33 specialists in educational technologies and/or in excess of infantile weight. The agreement index of 80% was the minimum considered to guarantee the validation of the material.

**Results:**

most of the specialists had a doctoral degree and a graduate degree in nursing. Regarding content, illustrations, layout and relevance, all items were validated and 69.7% of the experts considered the album as great. The overall agreement validation index for the educational technology was 0.88. Only the script-sheet 3 did not reach the cutoff point of the content validation index. Changes were made to the material, such as title change, inclusion of the school context and insertion of nutritionist and physical educator in the story narrated in the album.

**Conclusion:**

the proposed serial album was considered valid by experts regarding content and appearance, suggesting that this technology has the potential to contribute in health education by promoting healthy weight in the age group of 7 to 10 years.

## Introduction

Over the last three decades, the prevalence of excess weight in children, which includes overweight and obesity, has increased substantially, presenting itself as one of the great challenges of public health in the current context^1^.

This epidemiological and nutritional picture is explained, in large part, by the changes that have been occurring in the food pattern, resulting in more and more common obesogenic diets^2^. Add to this the reduction of physical activity and an increase in the practice of sedentary activities, such as watching television^3^. In addition, other factors associated with the etiology of obesity in children are: parental obesity, maternal diabetes, maternal smoking, gestational weight gain^4^, inadequate sleep^5^, influences of the family and school environment^6^, socioeconomic and educational aspects of the family^7^, among others.

This increase in infant excess weight is concerning, since in a study with children and adolescents aged 8 to 18 years, obese individuals, compared to eutrophic ones, were more likely to have hypertension, hyperinsulinemia, higher value of the homeostatic resistance assessment model (HOMA-IR), hypertriglyceridemia, low HDL-C, high LDL-C and increased uric acid^8^.

In addition to cardiometabolic outcomes, obesity in children has been associated with obesity in adulthood, mental health problems, asthma, obstructive sleep apnea, orthopedic difficulties, early maturation, polycystic ovary syndrome, and hepatic steatosis^9^.

In this context, interacting with children about habits that reduce the risk of obesity is relevant for health promotion and prevention of complications^9^. Thus, it is necessary to encourage the creation and use of educational technologies capable of mediating the interaction between health practitioners and children, aiming at the joint construction of knowledge about childhood obesity.

Associating care with educational actions aims to share practices and knowledge in a horizontal relationship. Thus, it is believed that the technologies are tools, processes or materials created to expand the possibilities of health practitioners to perform care practices and, consequently, improve the quality of care^10^.

Although it is relevant, in access to online search devices, there was a shortage of validated educational technologies on weight control and, mainly, having a target audience composed of Brazilian children. Thus, in view of the need to intervene in the problem of childhood obesity and to develop new technologies that are easily accessible and used by health and education professionals, a serial album was built with the purpose of empowering children from 7 to 10 years on the importance of maintaining healthy habits and, as a consequence, controlling body weight. 

At this juncture, it is worth emphasizing that, in order to give greater credibility and reliability to the educational materials that it is intended to implement, it is opportune to use an evaluation process to maximize their effectiveness^11^. In view of this, the present study was developed with the objective of validating the content and appearance of a serial album targeted at children aged 7 to 10 years addressing the topic of prevention and control of body weight. 

## Method

This is a methodological study, of descriptive character, through the opinion of a group of specialists. The process of construction of the serial album took place between the months of April and August of 2014 and the validation by specialists occurred between September and October of the said year.

The first version of the serial album was entitled “Excess weight in children” and, for the construction thereof, part of the theoretical content on the subject was gathered. Thus, we used publications from the National Survey on School Health (PeNSe)^12^ ^-^ ^13^ and from the World Health Organization^8^ ^,^ ^14^.

In addition, the content was planned based on the reality of health education, assistance and research with children and adolescents accompanied at the Center for Childhood Obesity (COI), Campina Grande/Paraíba, in the Northeast region of Brazil^5^ ^,^ ^15^.

The album was written according to the story of the Silva family whose set of characters was composed of two children (Maria and Francisco), the father (Seu José), the mother (Dona Lúcia), the grandmother (Dona Carminha) and the nurse Ana. These names were selected because they were considered common among the Brazilian population. In addition to the narration of the story, other pictures were inserted that served as a script for the dialogue between the children and the artist’s serial album.

The illustrations were elaborated by a designer, who used Adobe Ilustrator® and Corel Draw® to edit the images, and another specialized professional was responsible for the layout of the serial album through Photoshop®.

**Figure 1:**
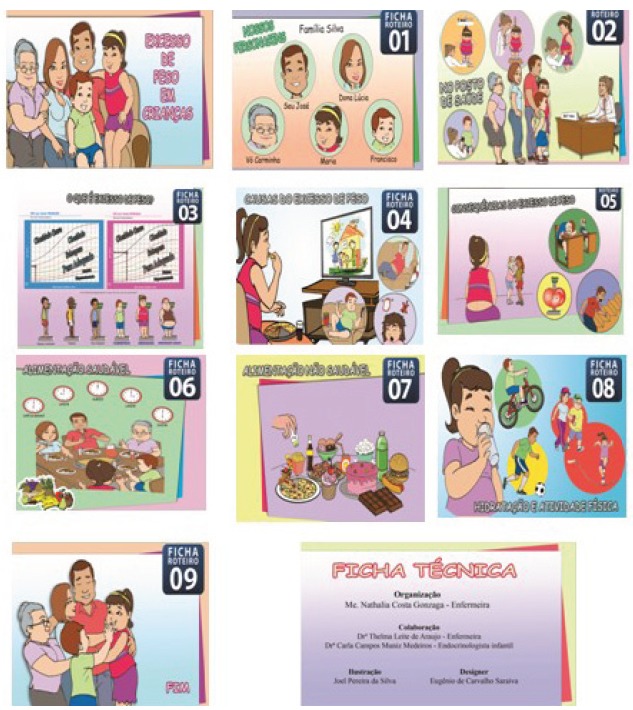
Cover page, figures and technical file of the first version of the serial album, titled “Excess weight in children”. Fortaleza, CE, Brazil, 2014

**Figure 2:**
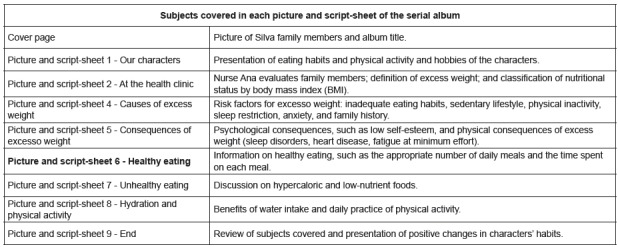
Subjects covered in the pictures and script-sheets of the first version of the serial album titled “Excess weight in children”. Fortaleza, CE, Brazil, 2014

In the end, the serial album consisted of 20 pages: cover page, nine pictures and the respective nine script-sheets and a technical file with the names of the makers (PhD student, advisors, illustrator and designer). Figure 1 shows the cover and the pictures of the first version of the serial album, besides the technical file. Figure 2 shows the summary description of the contents of the cover, the figures and the script-sheets of the serial album.

After being built, the validation of the serial album was carried out by a group of 33 specialists with extensive experience in the area of educational technologies and/or in excess of infantile weight, composing a multidisciplinary body. The criteria for selecting the specialists were: having, at least, the Master degree; having at least one publication in the area of elaboration and validation of educational technologies (serial album, booklet, video) and/or in the area related to excess weight in children and, among those who had published only in the area of child excess weight, having worked for at least one year in assistance in this area.

The sample size was defined by a formula that considers the final proportion of subjects in relation to a given dichotomous variable and the maximum acceptable difference of this proportion: n = Zα²×P×(1-P)/d², in which Zα refers to the confidence level adopted, which was 95%, P is the minimum proportion of individuals who agree with the pertinence of components of the serial album, considering 85%, and d is the difference of proportion considered acceptable, which was 15%. Thus, the final calculation was determined by n=1.96²×0.85×0.15/0.15², which resulted in approximately 22 specialists^16^. 

The survey of eligible specialists was done in the Lattes Platform of the CNPq portal and in the Database of Thesis of CAPES (Coordination of Improvement of Higher Level Personnel), using the following keywords: “obesity”, “overweight”, “ childhood obesity” and “educational technology”. Then, invitation letters were sent to the possible experts by e-mail, also asking the indication of other participants that met the selection criteria, resulting in some having been selected by snowball sampling, which is a method of sampling that is useful for studying difficult-to-access or difficult-to-study populations for which there is no precision about their quantity, which is the case of this study^17^. The Lattes Curriculum of the professionals indicated were analized to verify compliance with the inclusion criteria. 

A total of 79 experts from different regions of the country were invited to address food diversity and other habits, customs and cultural contexts. Of these, 17 did not return the contact, eight did not agree to participate in the study and 21, although having accepted the invitation, did not send the instrument completely filled within the stipulated time. Thus, the final sample consisted of 33 specialists.

The following documents were forwarded to the experts: a Free and Informed Consent Form (FICFs) in two copies, the first version of the serial album entitled “Excess weight in Children”, and the data collection instruments.

Data collection was performed using two instruments: one for characterization of the specialists and the second one called the Serial Album Analysis Protocol, organized in two sections. The first section was adapted from another study that also evaluated a serial album^18^ and was related to the appearance of the cover page and the pictures and the internal content of each script-sheet; while the second, also adapted^19^, was about evaluating the album as a whole. This section contained evaluative items of the material (content, illustrations, layout and relevance) answered in the form of Likert scale, in which TD = totally disagree; D = disagree; A = agree; and TA = totally agree. Also, there was a question regarding the general opinion about the album.

The instruments were adapted^18^ ^-^ ^19^ because they addressed other themes and, in the case of the second section, because it is a type of technology different from a serial album, necessitating, therefore, adjustments in some evaluative items. Both original instruments were not submitted to a scientific validation process.

The data were double typed in an electronic database and, after the consistency analysis, a descriptive study was carried out to characterize the specialists and their respective analyzes. The data are presented by means of proportions, means and standard deviations.

In order to analyze the content validity of the script-sheets, we used the Content Validity Index (CVI), calculated through two mathematical equations: the I-CVI (item-level content validity index) and the S-CVI/Ave (scale-level content validity index)^20^. In this study, I-CVI was defined as the content validity index of the individual items, calculated from the division between the number of positive responses to a given criterion of validation of the serial album on the total number of responses to the item, whereas S-CVI/Ave is understood as the mean of the content validation indexes for a given set of validation criteria of the serial album. Finally, we calculated the S-CVI Global (global content validity index) of the serial album, which represents the average of the I-CVIs for all the validation criteria of the serial album, according to the evaluations of the 33 specialists.

For the validation criteria of the serial album, the answers “clear”, “yes”, “relevant” and “very relevant” were classified as positive for the questions related to the assessment of the cover page, pictures and texts of the script-sheets; “I agree” and “I totally agree” for the group of questions about content, illustrations, layout and relevance; and “Good” and “Great” were considered positive responses in the question on the general opinion question about the album.

It should be noted that the CVI ranged from 0 to 1 and the serial album would be considered valid if it presented a S-CVI Global greater than or equal to 0.80^21^. The items that obtained percentage below 80% of agreement were reformulated based on the suggestions of the experts and the scientific literature.

The study was approved by an ethics committee according to Opinion No. 751,174. All norms for research with human beings, present in Resolution 466/2012 of the National Health Council of Brazil, were fulfilled. For preserving anonymity, experts were identified by the letter ‘E’ followed by a number ranging from 1 to 33, resulting in codes from E1 to E33.

## Results

The majority of the experts in this study were female (97.0%), had a doctoral degree (63.7%) and worked professionally in the Northeast (51.5%), Southwest (21.2%), South (21.2%) and North (6.1%) regions of Brazil. The mean age was 38.3 years (± 10.38), with 24 being the minimum and 60 the maximum age.

With regard to professional training, 60.6% were nurses and 30.3% were nutritionists. The lowest participation was from professionals of medicine, physical education and psychology, being only one of each. The average length of professional training was 15.42 years (± 10.0). The average experience time, in years, in the areas related to the object under study was 11.1 (± 8.5) in health education and 7.7 (± 5.8) in excess of child weight.

In addition, 84.8% of the experts had experience with child health, 60.6% in validation of educational material and 69.7% had already participated in validation of educational technology.

Among the eight professionals who did not accept to evaluate the serial album, six justified personal reasons and two did not find that the prevention of excess weight in children could be a topic of interest and nursing intervention, even refusing the invitation with this justification.

Table 1 shows the S-CVI/Ave of the cover page, the nine pictures and the nine script-sheets of the serial album. The set of picture and script-sheet 8 was considered the best. The script-sheet 2 and picture 9 had the mean exactly at the limit. Only script-sheet 3 did not reach the cut-off point.

**Table 1:** Distribution of the averages of content validation indexes (S-CVI/Ave) of the cover page, pictures and script-sheets of the serial album on control of child body weight according to the analysis of the specialists. Fortaleza, CE, Brazil, 2014

	Evaluation from specialists	
	Pictures S-CVI/Ave^*^	Scrip-sheets S-CVI/Ave^*^
Cover	0.83	
1	0.95	0.92
2	0.85	0.80
3	0.87	0.74
4	0.90	0.92
5	0.83	0.88
6	0.90	0.88
7	0.92	0.93
8	0.97	0.93
9	0.80	0.89
Mean	0.89	0.87

* S-CVI/Ave: Content validation indexes average

Some experts considered that script-sheet 3 had a difficult-to-understand content for children aged 7 to 10 years due to the presentation of the World Health Organization chart for the classification of body weight by the z-score of individuals aged 5 to 19 years^22^. For this reason, the chart was removed from picture 3 and script-sheet 3.

On the script-sheet 2, the main point discussed by the experts was related to the Body Mass Index (BMI), which presented a text that may be incomprehensible to children. Thus, the text on BMI was excluded. 

In the case of picture 9 of the serial album, some experts have suggested different endings of the story, such as: *finishing with the image of Maria leaner* (E1); I think I would put the family doing an activity - perhaps walking or playing with a ball (E6).

Concerning the presentation of the story involving a family, some experts presented their opinions: *due to the change in the structure of the families, I do not think this type of approach is adequate.* (E22); *I suggest removing the mention of the family ... because the theme and focus are the children.* (E12).

The percentage of specialists who considered the title of the first version of the serial album as appropriate was 69.7%. Three experts argued that, possibly, the term “excess weight” was not understandable for children (E30, E32 and E1). Aiming to meet the suggestions of the experts, the second version of the serial album was entitled “An Eye on Weight.”

Regarding content, illustrations, layout and relevance, all items were validated by specialists, presenting I-CVI higher than 0.80. Among the four blocks, the Relevance was the one with the highest S-CVI/Ave (Table 2).

**Table 2:** Level of agreement of the experts regarding the content, illustrations, layout and relevance of the serial album on control of chil body weight in children from 7 to 10 years. Fortaleza, CE, Brazil, 2014

General Evaluation Variables of the Serial Album	Level of agreement of experts				I-CVI^||^
	TD*	D^†^	A^‡^	TA^§^	
A. CONTENT					
A.1 Content is appropriate for the audience	0	4	16	13	0.88
A.2 Content is enough to meet the audience needs	0	6	16	11	0.82
A.3 The sequence of the text is logical	0	1	13	19	0.97
S-CVI/Ave^¶^ Content					0.89
B. ILLUSTRATIONS					
B.1 The illustrations are relevant to the content of the material and elucidate the content	1	4	12	15	0.82^**^
B.2 The illustrations are clear and convey easy comprehension	1	2	16	13	0.88^**^
B.3 The number of illustrations is appropriate for the content of the educational material	1	0	8	24	0.97
S-CVI/Ave^¶^ Ilustrações					0.89
C. LAYOUT					
C.1 The font used makes it easy to read	0	4	10	19	0.88
C.2 The colors applied to the text are pertinent and facilitating for Reading	0	3	10	20	0.91
C.3 The visual composition is attractive and well organized	1	2	14	16	0.91
C.4 The size of the title and text letters is adequate	1	2	11	19	0.91
S-CVI/Ave^¶^ Layout					0.90
D. RELEVANCE					
D.1 The themes portray key aspects that need to be strengthened	0	0	10	23	1.00
D.2 The material allows the transfer and generalizations of the learning to different contexts (school, home, health unit)	1	0	14	18	0.97
D.3 The material proposes the learner to acquire knowledge to prevent and control excess weight	1	4	9	19	0.85
D.4 The serial album is applicable in the daily life of health practitioners	1	0	9	23	0.97
S-CVI/Ave^¶^ Relevance					0.95

* TD: I totally disagree. ^†^D: I disagree. ^‡^A: I agree. ^§^TA: I totally disagree. ^||^I-CVI: Content Validity Index of Individual Items. ^¶^S-CVI/Ave: Content validation indexes average. ^**^ One expert did not answer to that question. The I-CVI calculation was based on the number of respondents.

In the layout block, some experts suggested that the red color should be avoided in the letters of the script-sheets, and in order to improve visibility, the red color was replaced by dark green.

Among the specialists, 69.7% considered the album to be excellent, 27.3%, good and only 3.0% stated that the technology was of poor quality. The S-CVI/Ave Global of the educational technology was 0.88, confirming the validation of appearance and content by specialists.

**Figure 3:**
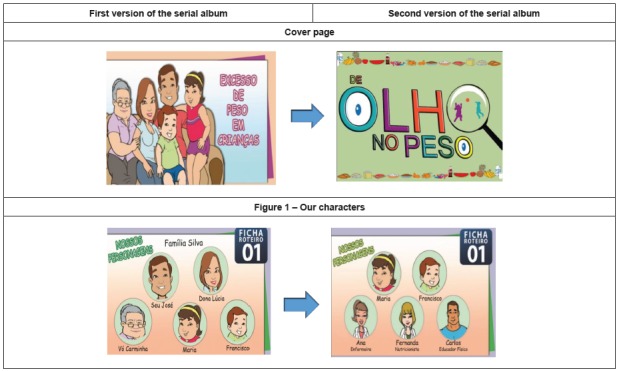
Versions 1 and 2 of the cover page and illustration of the characters of the serial album story. Fortaleza, CE, Brazil, 2014

After reflecting on the suggestions of the experts, the illustrator and the designer were again contacted in order to develop changes in the pictures and texts, thus obtaining the second version of the serial album. Figure 3 shows the two alterations made, respectively, in the cover page and in the illustration of the characters of the story of the serial album.

## Discussion

In this study, the album “An Eye on the Weight” on child body weight control was validated by specialists with extensive experience in the area of educational technologies and/or excess child weight. This educational material represents a technological innovation in Brazil, considering that, although the topic of childhood obesity has been much discussed, few validated technologies were found addressing this theme, especially with children as a target audience. 

It is emphasized that the serial album is a technology that is easy to use in health services and schools, because it is classified as independent, that is, it does not depend on electrical resources for use^10^. In addition, a positive feature of this album was the creation of characters and the narration of a story, which allows a playful approach to the theme.

This playful aspect was also focused by another Brazilian study, in which the authors developed a serious game for schoolchildren with the topic of human digestion, healthy eating and physical exercise and obtained a positive evaluation from a group of health and computer specialists^23^. In addition to this game, a smartphone application was found that aims to promote healthy behaviors among children; this was elaborated by researchers from South Korea^24^. 

The elaboration of different health technologies assists professionals, who can take advantage of them as a way to assist their clientele and promote autonomy and independence, whether in closed institutions, in education in health or in any environment^25^.

Although the educational approach of the serial album validated in this study was mainly the traditional one, in which the educator explains to the student healthy habits and behaviors^26^, it is emphasized that the application of this material aims to stimulate dialogue with children through questions about individual habits and opinions about the story. 

On the theme of the serial album, it is believed that, in order to restraint the growing tendency of excess weight, interventions should start in childhood^14^. Thus, aiming at promoting the health of all children, regardless of nutritional classification, the purpose of the educational material was redefined, which came to focus on raising awareness of the target audience about the importance of taking care of body weight through healthy habits and not only highlighting obesity. Thus, the serial album came to be entitled “An Eye on Weight”, and not “Excess weight in children”.

In the validation process, the evaluation of the specialists was of paramount importance for the improvement of the material and it is valid to highlight the scope of a professional diversity, resulting in a multidisciplinary work.

In this context, it should be emphasized that the factors related to weight changes are not limited to feeding and, therefore, this reaffirms the importance of a multidisciplinary follow-up focusing on the establishment of healthy habits, related to diet and practice of physical activity associated to the provision of psychological support^27^.

However, two professionals not trained in nursing refused the invitation to be part of the group of specialists in this study because they consider that the prevention of excess weight in children is not an object of nursing intervention, which is the training area of the main developer of this technology education. This perspective of nursing, apart from issues related to body weight control, is shared even by some members of this professional category. A study found that primary health care nurses demonstrated, despite reaching a favorable level of knowledge about excess weight in adolescents, a posture in which they exempt themselves from great impact conducts, either in health promotion, prevention of illness or treatment, attributing to other professionals greater responsibility for this problem^28^.

However, it is worth noting that in the latest version of NANDA-I^29^, excess weight is configured as the following three nursing diagnoses: Obesity, Overweight and Risk for Overweight, reinforcing this problem as amenable to nursing prevention/identification/intervention.

In the analysis of the serial album, one of the main topics approached by the experts was the participation of a family in the story. In this context, although there is a strong influence of the family on the genesis of excess weight in children^30^, we decided not to address the family issue in the second version of the serial album because, according to the interpretation of some experts, the album could be conveying constructions of family nuclei different from the habitual ones of the children and generating possible conflicts.

In view of the exclusion of the family group, the school scenario was included, which is an important locus for the development of educational strategies in health with the purpose of developing the autonomy of children and adolescents. Furthermore, in Brazil, the Health in School Program (PSE) has contributed to establish a link between the professionals that integrate the Family Health Strategy and the school^31^.

Thus, this scenario change also resulted in the exclusion of characters (father, mother and grandmother) and the inclusion of two other health professionals: nutritionist Fernanda and physical educator Carlos. As already mentioned, body weight control includes the participation of professionals from different areas; besides these, there are physicians, psychologists, pedagogues, physiotherapists, occupational therapists, among others^27^. Nevertheless, the insertion of a more complete team could confuse the children participating in the application of the album, and they possibly would not understand the function of each professional.

Another change in the serial album was the union of pictures 6 (healthy eating) and 7 (unhealthy eating), and this happened because some experts suggested not labeling foods as ‘healthy’ and others as ‘unhealthy’, mainly because those ‘unhealthy’ foods might look very appetizing to the child’s eyes and the ‘healthy’ ones would look like bad tasting foods.

Finally, the picture and the content of the script-sheet 9 were changed and became the picture and sheet 8 in the second version of the serial album, constituting a review of the topics covered in the educational intervention. In this sheet, the children are invited to put into practice the guidelines received in the educational intervention: Be part of this team! Therefore, it is incumbent upon all professionals, school, society and parents to share knowledge with children by offering opportunities for the practice of healthy habits and thus awakening them to a critical vision and a transforming attitude in the face of the obesity epidemic. 

In addition to the health education activities that take place at the micro-scope level, it is necessary to understand that addressing childhood obesity requires a set of evidence-based multisectoral policies that enable environments conducive to healthy lifestyles. In a study on antiobesity policies in Latin America, some policies were cited, including: (i) special taxes on sugary drinks and high calorie foods; (ii) food labeling legislation; (iii) removal of trans fatty acids from processed foods; and (iv) recreational cycle paths or “open streets”^32^.

Regarding the limitations of this study, one of them may be the predominance of specialists from the Northeast region (51.5%), which may suggest a tendency to the habits of this region. However, it is noteworthy that 79 specialists from all regions of the country were invited and, from this, it was verified they had difficulty in returning the contact and in completing the instrument. In addition, it is worth mentioning that the sample size reached, which was 33 specialists, was much higher than the minimum required, which was 22.

Another possible limitation is the fact that the experts had not been questioned whether they had knowledge about education for children aged 7 to 10 years. Thus, in order to reduce this limitation, it is suggested as a future study an evaluation of the serial album by pedagogues.

Finally, the scientific advance in the area of health education of Brazilian children stands out, since the proposed serial album has been validated by specialists in content and appearance. Therefore, it is intended to contribute with health professionals in the process of raising children’ awareness in a playful and educational way regarding the risks associated with childhood obesity. As future studies, it is suggested the validation of the serial album by the target audience and the construction of another version with specific content for the parents and/or legal guardians for the children.

## Conclusion

The proposed serial album was validated by experts with experience in educational technologies and/or excess child weight with S-CVI Global equal to 0.88, which suggests that this technology can be used in health education activities with the aim of promoting healthy behaviors though the learning of skills related to body weight control.

We believe that this process of adapting the educational technology to the suggestions of the specialists was an essential step to make the material more appropriate to children and with greater scientific rigor.

Thus, the result of this research can be a relevant educational instrument for application in specific activities or in conjunction with a program of interventions to combat childhood obesity, either in health units or schools. It should be noted that, according to the validation made by specialists, the illustrations were considered clear, attractive and relevant, and the content was evaluated as easy to understand for children in the age group of 7 to 10 years. However, in order to improve this technology, it is suggested a further study for the evaluation of the album by the target audience. 
